# Bifunctional compounds for targeted degradation of carbonic anhydrase IX through integrin-facilitated lysosome degradation

**DOI:** 10.1016/j.jbc.2025.108482

**Published:** 2025-04-07

**Authors:** Wanyi He, Congli Chen, Runjie Cai, Jiwei Zheng, Mengyu Yao, Joong Sup Shim, Hang Fai Kwok, Xiaojun Yao, Lijing Fang, Liang Chen

**Affiliations:** 1 Institute of Biomedicine and Biotechnology, Shenzhen Institute of Advanced Technology, Chinese Academy of Sciences, Shenzhen, Guangdong, China; 2 School of Pharmacy, Changzhou University, Changzhou, Jiangsu Province, China; 3 Cancer Centre, Faculty of Health Sciences, University of Macau, Avenida da Universidade, Taipa, Macau SAR; 4 Centre for Artificial Intelligence Driven Drug Discovery, Faculty of Applied Sciences, Macao Polytechnic University, Macao; 5 University of Chinese Academy of Sciences, Beijing, China

**Keywords:** targeted protein degradation, carbonic anhydrase IX, integrin-facilitated lysosomal degradation, bifunctional small molecule drug, hypoxic

## Abstract

As an important therapeutic target, carbonic anhydrase IX (CAIX) is crucial in the pH regulation of hypoxic solid tumors, thus keeping the survival of them in acidic microenvironment and promoting their proliferation, invasion, and metastasis. To degrade endogenous CAIX, three bifunctional compounds were designed according to the integrin-facilitated lysosomal degradation strategy. These compounds are composed of a CAIX-binding ligand, an integrin-recognizing ligand, connected *via* a linker, which could induce CAIX degradation in an integrin- and lysosome-dependent manner. Among them, Sul-L1-RGD showed the highest degradation efficacy and could inhibit the proliferation of tumor cells under hypoxic conditions, thus it has great potential to be applied in cancer drug discovery.

As an emerging strategy for targeted protein degradation, proteolysis-targeting chimeras (PROTAC) has become one of the most attractive drug development technologies, which utilizes the ubiquitin-proteasome system (UPS) for specific recognition and effective degradation of target proteins (1, 2, 3). However, PROTACs are mainly applied to degrade intracellular proteins due to the limitation of the UPS (4, 5, 6). In addition, autophagosome-tethering compound (7) and autophagy-targeting chimera (8) utilize the autophagy-lysosome pathway to induce target proteins degradation by recruiting autophagosomes or binding to LC3. As a complement to above technologies, degradation of extracellular and membrane-associated proteins *via* endocytosis and lysosome pathways has attracted widespread attention in chemical biology and drug discovery, such as lysosome-targeting chimera (9, 10, 11), Bispecific aptamer chimera (12), antibody-based PROTAC (AbTACs) (13), ASGPR targeting chimeras (9, 11), covalent nanobody-based PROTACs (GlueTACs) (14), and other related technologies (15, 16). The protein degradation techniques mentioned above induce the internalization and degradation of the target proteins by binding the extracellular domain of the targeted proteins to the cell surface lysosomal targeting receptor, transmembrane E3 ligase, or lysosomal sorting peptide.

Previously, we reported a novel integrin-facilitated lysosomal degradation (IFLD) strategy for extracellular and membrane-associated proteins (17). Integrin is a heterodimer transmembrane protein receptor that mediates the connection between intracellular and extracellular matrix (18, 19). Some integrins such as α_v_β_1_, α_v_β_3_, and α_v_β_5_ can be recognized by arginine-glycine-aspartate (Arg-Gly-Asp, RGD) sequence of native extracellular matrix ligands (20, 21, 22). Therefore, RGD has been extensively used to deliver fluorescent groups, small molecule drugs, or nanomaterials into cells through integrin-mediated endocytosis. In our IFLD strategy, the designed bifunctional compounds are composed of a small molecule as the target protein-binding ligand, an RGD cyclopeptide as the integrin binding ligand, and a specially designed "Linker" that covalently links two active ligands together. It was demonstrated that this kind of bifunctional compounds could efficiently induce the endocytosis and degradation of some extracellular and membrane-associated proteins in an integrin- and lysosome-dependent manner. Since α_v_β_3_ and α_v_β_5_ integrins are widely expressed on the surface of various tumor cells, the IFLD strategy is especially useful in tumor target therapies. Therefore, we attempt to further expand the application of the IFLD strategy to degrade carbonic anhydrase IX (CAIX), which is a transmembrane protein that is overexpressed in progressive and invasive tumors, such as colon cancer, lung cancer, and breast cancer The expression of CAIX is hypoxia-dependent and regulated by hypoxia-inducible factor (23, 24). It is well known that hypoxia and acidic microenvironment are the main characteristics of solid tumors. CAIX catalyzes the conversion of carbon dioxide hydrates into bicarbonate and protons, thus participating in the pH regulation of hypoxic solid tumors (25, 26). It plays an important role in keeping the survival of tumor cells in acidic microenvironment and promoting the proliferation, invasion, and metastasis of them, thus has been considered as an important therapeutic target (27, 28). Many inhibitors of CAIX have been developed, and SLC-0111 has reached the clinical trial. Therefore, we envisioned that a bifunctional compound that could specifically target and degrade CAIX would be very valuable in the treatment of solid tumors.

Herein, to degrade CAIX, we designed some novel bifunctional compounds, containing a ligand that can specifically recognize CAIX and a cyclic RGD peptide that can specifically bind to integrin, connected *via* a linker. Among them, Sul-L1-RGD was found to be effective in inducing CAIX degradation and inhibiting the survival of tumor cells during hypoxic conditions (Fig. 1).

**Figure 1:**
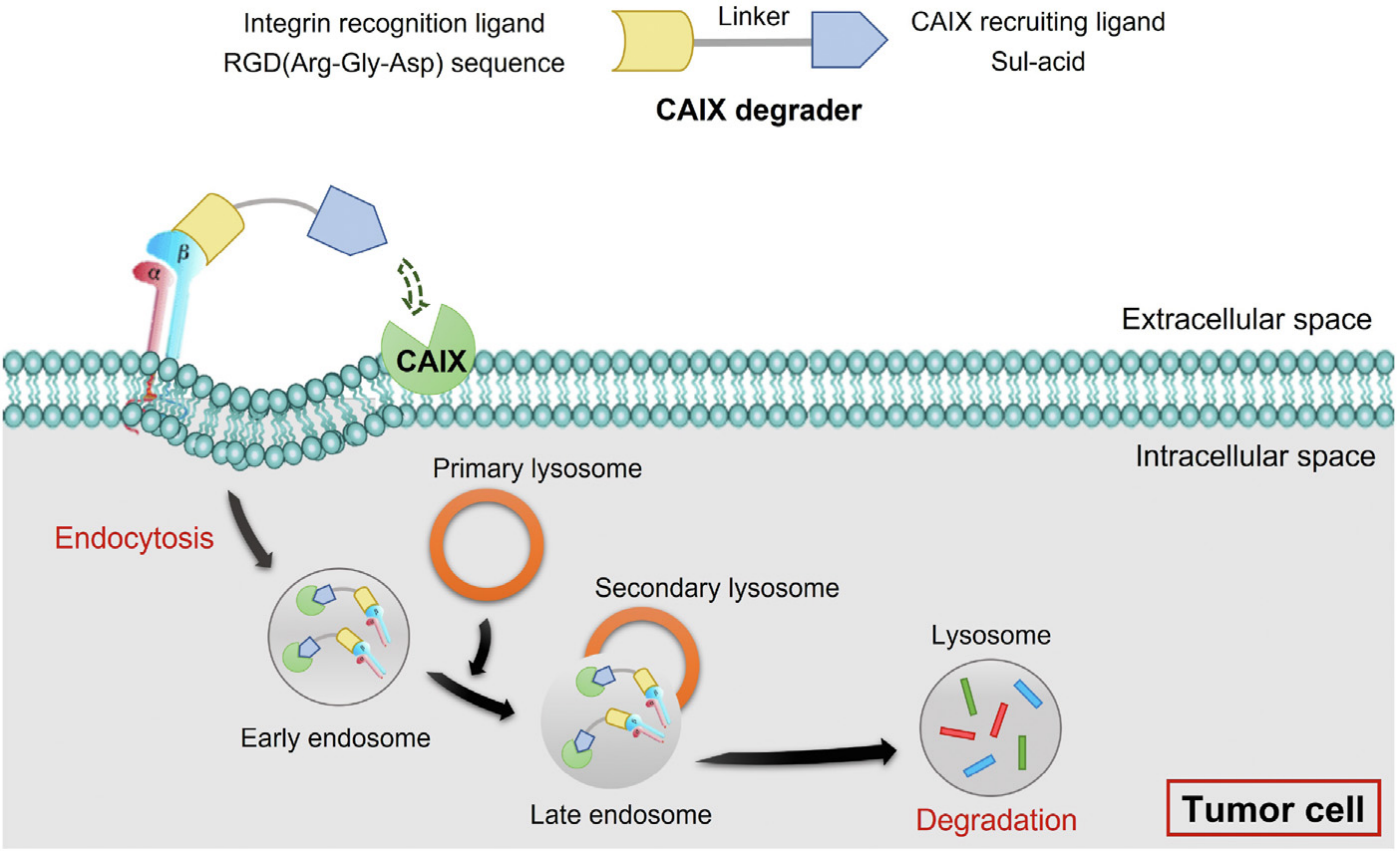
**Schematic representation of the study.** Bifunctional compounds mediate degradation of carbonic anhydrase IX localized on cell membrane through integrin-facilitated lysosome degradation. CAIX, carbonic anhydrase IX.

## Results

### Design and synthesis of CAIX degraders

**Figure 2:**
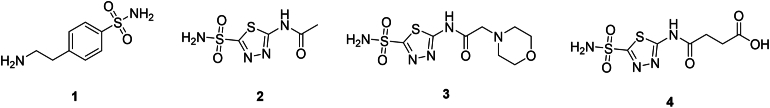
**Small molecule inhibitors of CAIX**.

It has been reported that the main pharmacodynamic group of CAIX inhibitors is the sulfonamide group, which can target Zn^2+^ in the active center of CA to form coordination bonds and interfere with the function of the enzymes. For example, the sulfonamide compounds 1 to 4 have good inhibitory activity toward CAIX (Fig. 2). Among the small molecule inhibitors targeting CAIX with high affinity, we selected compound 4, 4-[5-(aminosulfonyl)-1,3,4-thiadiazole] amino-4-oxobutyric acid, as the small molecule ligand of CAIX in our strategy and named it as Sul-acid, which contains an acid group that readily reacts with a linker to connect with a cyclic RGD peptide.

According to the IFLD strategy, we designed and synthesized three compounds with different linker lengths for targeted degradation of CAIX, Sul-L1-RGD, Sul-L2-RGD, and Sul-L3-RGD. These compounds are prepared by click chemistry between RGD-alkyne and Sul-linker-Azide (Sul-L1-Azide, Sul-L2-Azide, and Sul-L3-Azide) in the presence of CuSO_4_/NaVC, and the purities of these compounds was detected by analytical HPLC as 97.81%, 97.01%, and 96.22%, respectively and structures were confirmed by HRMS and ^1^H NMR (Figs. S1–S10).

### Sul-L1-RGD has the highest degradation efficiency among a series of CAIX degraders

**Figure 3:**
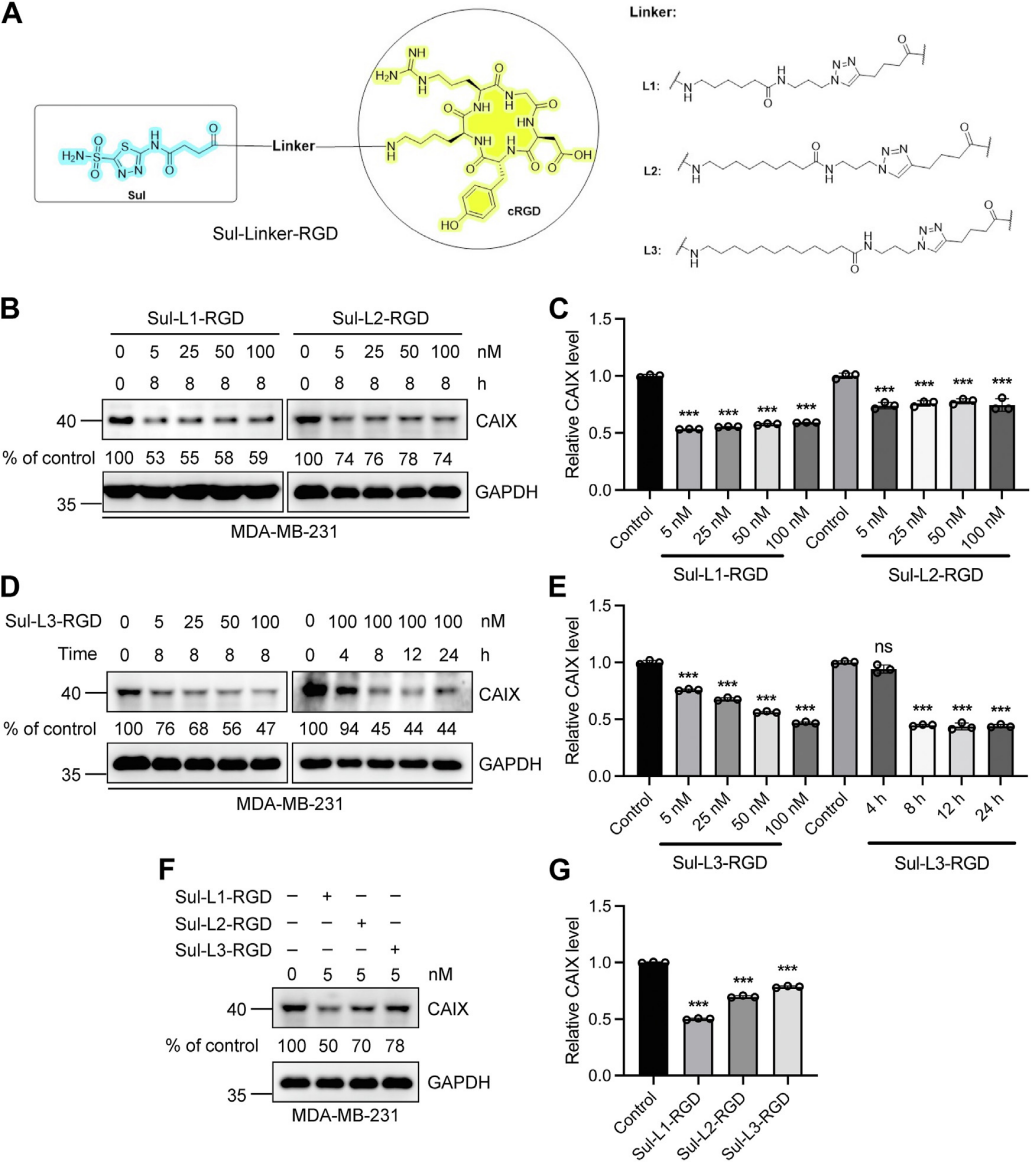
**Optimization of Sul-Linker-RGD "linker" structure to screen the best CAIX degrader.***A*, structure of Sul-L1-RGD, Sul-L2-RGD, and Sul-L3-RGD. *B* and *C*, Western blot analysis of CAIX degradation induced by Sul-L1-RGD or Sul-L2-RGD at different concentrations. MDA-MB-231 cells were treated with 5, 25, 50, and 100 nM Sul-L1-RGD or Sul-L2-RGD, respectively, for 8 h. *D* and *E*, Western blot analysis of Sul-L3-RGD–mediated CAIX degradation at indicated concentration or indicated time in MDA-MB-231 cells, respectively. *F* and *G*, Western blot analysis of the effects of different linker lengths on induced degradation of CAIX. *C*, *E*, and *G*, densitometry was used to calculate protein levels, and data were normalized to control. n = 3 biologically independent experiments; data are represented as mean values, and error bars represent the SD of biological replicates. The statistical significance was assessed using one-way ANOVA with Sidak's multiple comparisons test, ∗∗∗*p* < 0.001, n.s., not significant.

At the molecular level, the bifunctional compound Sul-linker-RGD can trigger the targeted degradation of CAIX on the premise that a ternary complex can be formed by CAIX, integrin, and the degrader on the cell membrane surface. Therefore, the length of the "linker" used to connect the CAIX-binding molecule Sul-acid and the integrin-recognized ligand RGD may affect the formation of the ternary complex, which would influence the degradation efficiency of CAIX. Therefore, we synthesized three bifunctional compounds with different linker lengths, including Sul-L1-RGD, Sul-L2-RGD, and Sul-L3-RGD (Fig. 3*A*). Then the degradation of CAIX mediated by these compounds was studied. Human breast cancer cell line MDA-MB-231 with both high expression level of CAIX and integrin was treated with Sul-L1-RGD, Sul-L2-RGD, and Sul-L3-RGD, respectively. The results showed that all three compounds with different chain lengths could induce the degradation of CAIX, among which the most effective degradation was observed when Sul-L3-RGD was used at the concentration of 100 nM (Fig. 3, *B*–*E*). Meanwhile, a time-course experiment was performed to evaluate the time-dependent degradation of CAIX and 8 h was revealed as the optimal duration (Fig. 3, *D* and *E*). However, Sul-L1-RGD showed better degradation efficiency compared with the other two compounds at a lower concentration of 5 nM (Fig. 3, *F* and *G*). To diminish the potential off-target effects caused by high dosage, Sul-L1-RGD was selected for subsequent experimental study.

### Sul-L1-RGD can effectively degrade CAIX through lysosome pathway *in vitro*

**Figure 4:**
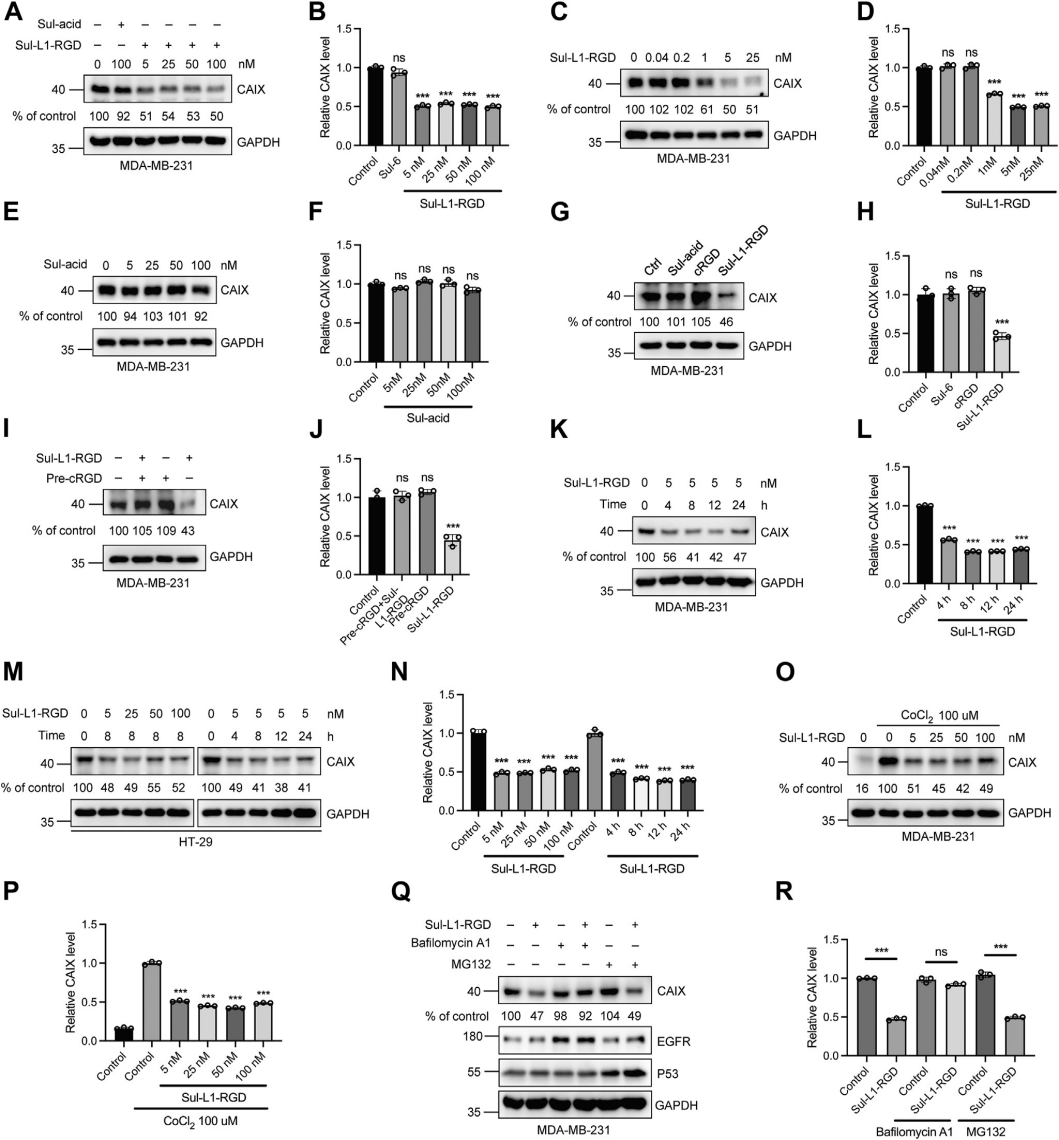
**Sul-L1-RGD degrades CAIX *via* the lysosomal pathway.***A* and *B*, Western blot analysis of CAIX degradation induced by Sul-L1-RGD at different concentrations. *C* and *D*, Western blot analysis of the differences in inducing CAIX degradation after treatment with several lower concentrations (ranging from 0.04 nM to 25 nM) of Sul-L1-RGD for 8 h *E* and *F*, Western blot analysis of CAIX degradation induced by different concentrations of Sul-acid. MDA-MB-231 cells were treated with 5, 25, 50, and 100 nM of Sul-acid for 8 h *G* and *H*, Western blot analysis of CAIX levels in MDA-MB-231 cells treated with Sul-acid, cRGD, and Sul-L1-RGD at 5 nM for 8 h. *I* and *J*, effects of competitive inhibition of cRGD-integrin binding on Sul-L1-RGD–mediated CAIX degradation, analyzed by Western blot. *K*–*N*, Western blot analysis of Sul-L1-RGD–mediated CAIX degradation at indicated concentration or indicated time in MDA-MB-231 (*K* and *L*) and HT-29 (*M* and *N*) cells, respectively. *O* and *P*, Western blot analysis of CAIX degradation induced by Sul-L1-RGD at different concentrations in the presence of 100 μM cobalt chloride solution. *Q* and *R*, Western blot analysis of MDA-MB-231 cells treated with 5 nM Sul-L1-RGD for 8 h along with 100 nM bafilomycin A1 or 5 μM MG132. *B*, *D*, *F*, *H*, *J*, *L*, *N*, *P*, and *R*, densitometry was used to calculate protein levels, and data were normalized to control. n = 3 biologically independent experiments; Data are represented as mean values, and error bars represent the SD of biological replicates. The statistical significance was assessed using one-way ANOVA with Sidak's multiple comparisons test, ∗∗∗*p* < 0.001, n.s., not significant.

To exclude the possibility that Sul-acid can induce the degradation of CAIX, MDA-MB-231 cells were treated with this small molecule and Sul-L1-RGD, respectively. It was shown that Sul-L1-RGD exhibited similar degradation effect at 5 to 100 nM, with minimal impact observed below 1 nM (Fig. 4, *A*–*D*). Few degradation of CAIX was observed in Sul-acid–treated group even at concentrations ranging from 5 nM to 100 nM, suggesting that Sul-acid does not lead to the degradation of CAIX by itself (Fig. 4, *E* and *F*). Similarly, cRGD could not induce the degradation of CAIX under the same conditions (Fig. 4, *G* and *H*). To test if the degradation of CAIX was mediated by *α*v*β*3 integrin, MDA-MB-231 cells were pretreated with an excess amount of cRGD to coat the integrins on cell surface and then incubated with Sul-L1-RGD. As expected, Sul-L1-RGD treatment did not induce CAIX degradation under this condition (Fig. 4, *I* and *J*). Then, the incubation time was further screened; the optimal incubation time for Sul-L1-RGD–mediated CAIX degradation was between 8 to 12 h, during which time more than 50% of CAIX proteins were degraded (Fig. 4, *K* and *L*). In addition, human colon cancer cell line HT-29 with high expressing level of both CAIX and RGD was treated with Sul-L1-RGD at different drug concentrations and different incubation times. Consistent with MDA-MB-231 cells, half of CAIX in HT-29 cells was degraded by Sul-L1-RGD at 5 nM in 8 h, indicating that Sul-L1-RGD has the capability to degrade CAIX in multiple tumor cells (Fig. 4, *M* and *N*).

CAIX acts as an endogenous marker of cellular hypoxia, and its expression is induced by the hypoxia signal present in most solid tumors (29). CoCl_2_ is an iron chelator that can replace iron ions in deoxyhemoglobin, preventing hemoglobin from binding to oxygen and causing intracellular hypoxia, which upregulates CAIX protein expression (30). To determine the potency of Sul-L1-RGD to degrade CAIX under hypoxia, MDA-MB-231 cells were pretreated cobalt dichloride (CoCl_2_) to create a hypoxia environment and then treated with varying concentrations of Sul-L1-RGD. The increased expression of CAIX protein in the CoCl2-treated group compared to the untreated group indicates the successful induction of hypoxia in tumor cells. It was shown that Sul-L1-RGD also exhibited significant degradation effect at 5 to 100 nM under this hypoxia condition (Fig. 4, *O* and *P*).

Currently, targeted protein degradation technologies mainly degrade target proteins through the ubiquitin-proteasome pathway or lysosome pathway. In order to explore the degradation mechanism of CAIX mediated by Sul-L1-RGD, MDA-MB-231 cells were incubated with Sul-L1-RGD in the presence or absence of lysosome inhibitor bafilomycin A1 or the proteasome inhibitor MG132, respectively. The results showed that the degradation of CAIX could be attenuated by lysosomal inhibitors rather than proteasome inhibitors, demonstrating that the degradation of CAIX was dependent on the lysosomal pathway (Fig. 4, *Q* and *R*). Changes in the expression of EGFR protein were provided as a reference for bafilomycin A1 treatment. In MG132 treated group, the level of P53 protein was detected as a positive control (Fig. 4*Q*) (31). In conclusion, Sul-L1-RGD can effectively degrade CAIX through the lysosome pathway.

### The degradation of CAIX by Sul-L1-RGD is mediated by **α**_v_**β**_3_ integrin

**Figure 5:**
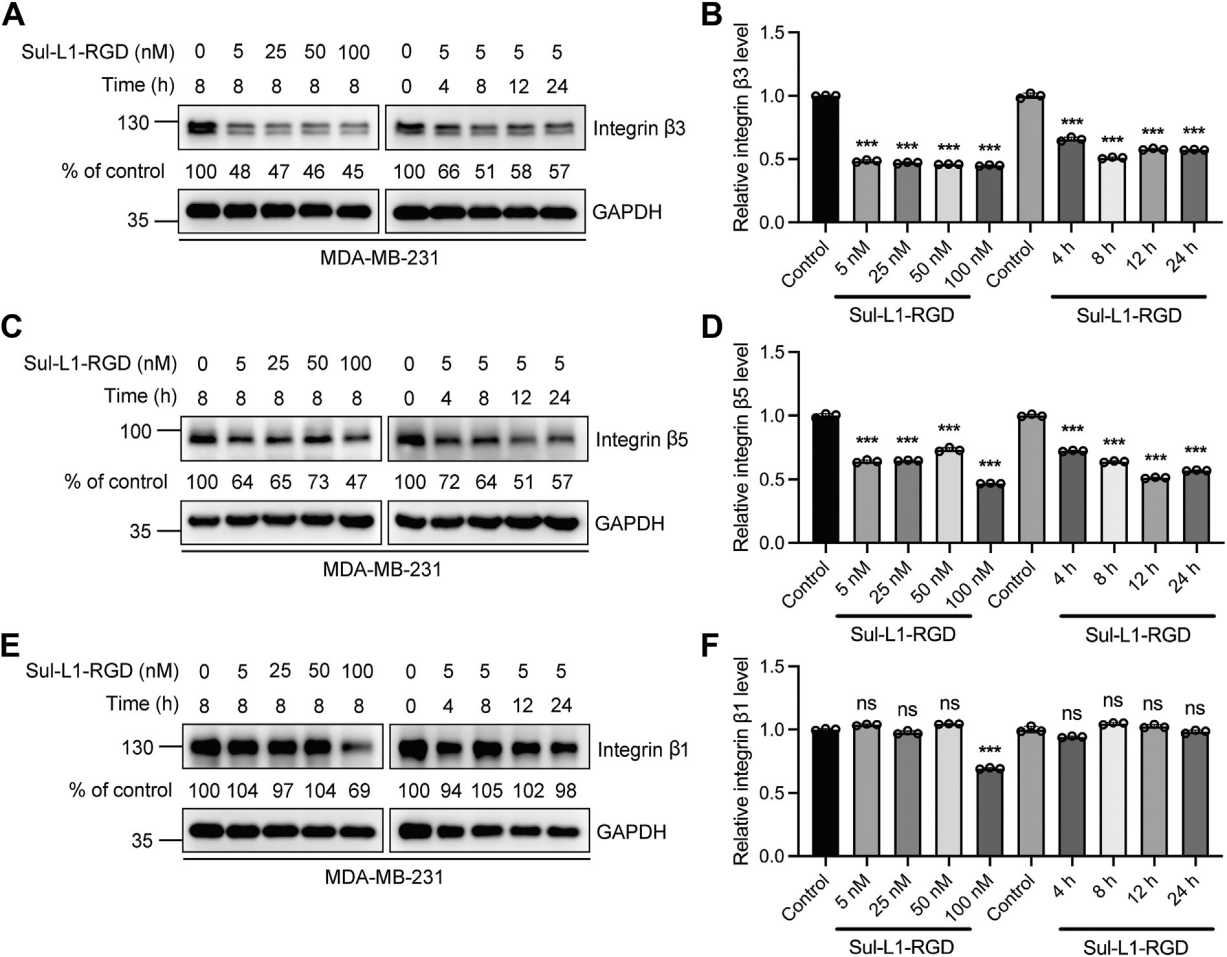
**Sul-L1-RGD degrades CAIX mainly through αvβ3 integrin.** Western blot analysis of Sul-L1-RGD-mediated integrin β3 (*A* and *B*), integrin β5 (*C* and *D*) and integrin β1 (*E* and *F*) degradation at indicated concentration, or indicated time. (*B*, *D*, and *F*) Densitometry was used to calculate protein levels, and data were normalized to control. n = 3 biologically independent experiments; Data are represented as mean values, and error bars represent the standard deviation of biological replicates. The statistical significance was assessed using one-way analysis of variance (ANOVA) with Sidak's multiple comparisons test, ∗∗∗*p* < 0.001, n.s., not significant.

Integrin is a divalent cation-dependent heterodimer transmembrane glycoprotein composed of two noncovalentally-related α and β subunits, including 18 α subunits (α1-α11, αv, αL, αE, αM, αX, αD, αⅡb) and 8 β subunits (β1-β8) composed of 24 different heterodimer integrin proteins. As the integrin recognition ligand, cyclic RGD bearing the Arg-Gly-Asp sequence has high affinity and specificity to α_v_β_3_ integrin while maintaining relative selectivity to α_v_β_5_ integrin. In the previous work of our group, we have verified that RGD–integrin interaction induced the endocytosis and degradation of the target protein in the IFLD strategy (17). In this work, we further evaluated whether the target protein degradation was mediated by α_v_β_3_ integrin. MDA-MB-231 was incubated with Sul-L1-RGD at different drug concentrations for different times. And the level of α_v_β_1_, α_v_β_3_, and α_v_β_5_ integrin was detected by Western blot analysis. As expected, about 50% reduction of α_v_β_3_, 36% reduction of α_v_β_5_, and 6% reduction of α_v_β_1_ were observed in MDA-MB-231 cells which were treated with 5 nM Sul-L1-RGD for 8 h, the optimal condition used to degrade CAIX (Fig. 5, *A*–*F*). These results indicated that the degradation of the target protein was mainly facilitated by α_v_β_3_ integrin; α_v_β_5_ was also involved in this process, while α_v_β_1_ was hardly impacted. It is known that α_v_β_3_ integrin is overexpressed in a variety of tumor cells and plays an important role in tumor metastasis and invasion and thus has received extensive attention in tumor-targeted therapy. Therefore, it is anticipated that Sul-L1-RGD has high selectivity to tumor cells since it can target both CAIX and α_v_β_3_ integrin on the cell membrane and induce the degradation of them simultaneously.

### Sul-L1-RGD can inhibit the survival of hypoxic tumor cells

**Figure 6:**
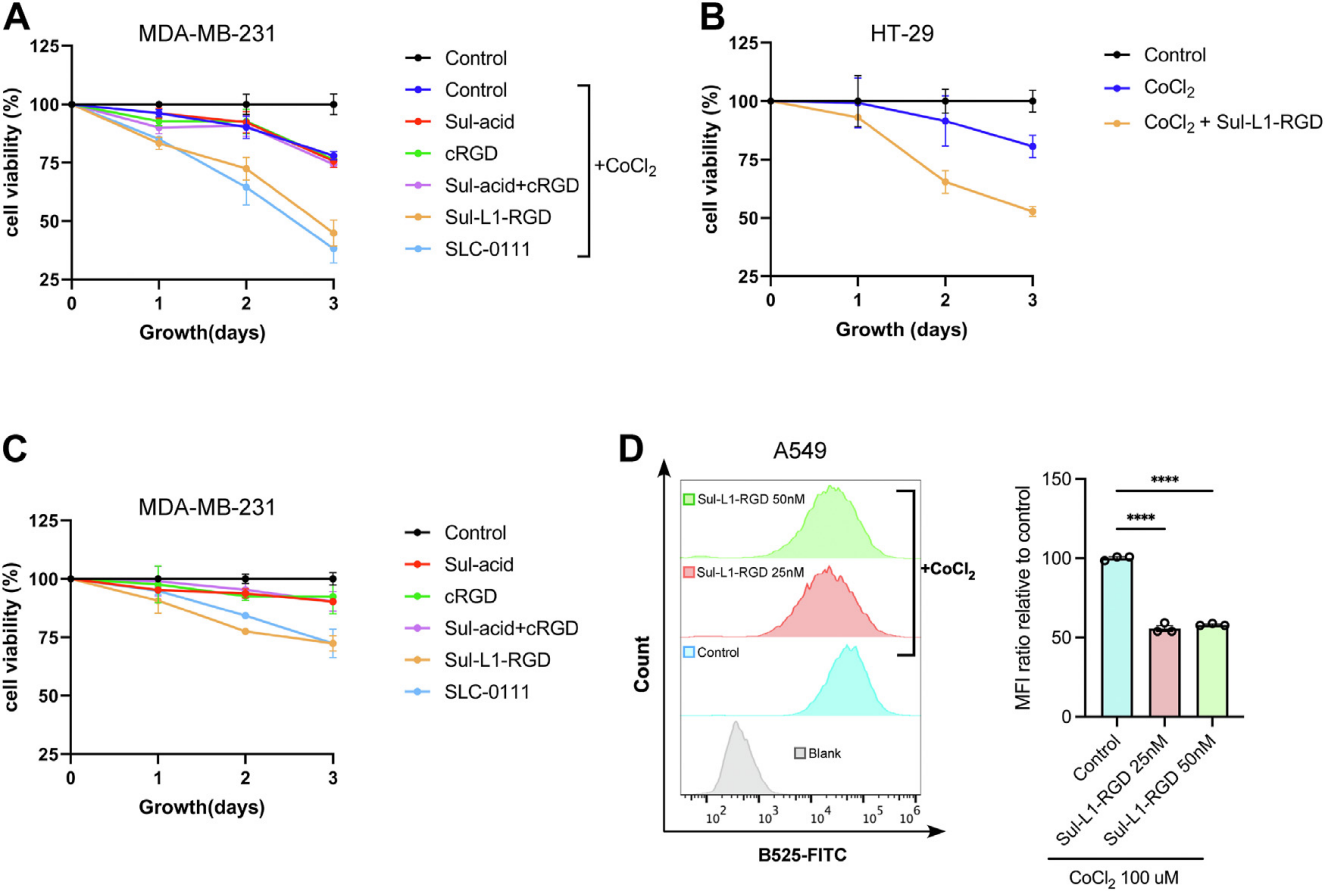
**Sul-L1-RGD can inhibit the survival of hypoxic tumor cells.***A* and *B*, the effect of Sul-L1-RGD treatment on survival of MDA-MB-231 (*A*) and HT-29 (*B*) cells under hypoxia condition analyzed by CCK8. *C*, the effect of Sul-L1-RGD treatment on survival of MDA-MB-231 cells under normoxia condition analyzed by CCK8; n = 3 biologically independent experiments. Data represent the mean ± SD. *D*, the effect of Sul-L1-RGD treatment on the changes in intracellular pH in A549 cells by the pH-sensitive fluorescent probe BCECF AM (5 μM). Densitometry was used to calculate average fluorescence intensity, and data were normalized to control. n = 3 biologically independent experiments; data are represented as mean values, and error bars represent the SD of biological replicates. The statistical significance was assessed using one-way ANOVA with Sidak's multiple comparisons test, ∗∗∗∗*p* < 0.0001, n.s., not significant.

It is well known that CAIX catalyzes the hydration of carbon dioxide into hydrogen ions and bicarbonate, thus regulating the acid-base balance inside and outside the cell, which is a key factor for the survival of cancer cells. When this process was blocked by the degradation of CAIX on the membrane, it would destroy the acid-base balance and also led to the decrease in bicarbonate, a substance necessary for proteins and nucleotides synthesis, which affects the metabolic pathways of cancer cells. In order to explore the inhibitory ability of Sul-L1-RGD on cancer cells under hypoxic conditions, HT-29 and MDA-MB-231 cells were treated with 5 nM Sul-L1-RGD in the presence of 100 μM cobalt dichloride solution. As illustrated by cell viability data (Fig. 6, *A* and *B*), Sul-L1-RGD could significantly inhibit the survival of tumor cells under hypoxia conditions, and the inhibitory activity of Sul-L1-RGD was comparable to that of SLC-0111, a CAIX inhibitor in phase Ib/II clinical trials (Fig. 6, *A* and *C*). In addition, Sul-acid or cRGD did not affect the survival of cancer cells either alone or in combination in both hypoxia and normoxia (Fig. 6, *A* and *C*).

### Sul-L1-RGD can reduce the intracellular pH of tumor cells

CAIX catalyzes the conversion of external carbon dioxide into bicarbonate and hydrogen ions, with the bicarbonate transported across the plasma membrane into the cytosol (32). This CAIX-mediated ion exchange mechanism ensures that lactate and protons, generated by the rapid glycolysis fueling actively proliferating tumor cells, are effectively neutralized by HCO_3_^-^, thereby preventing excessive intracellular acidification or acidosis. To investigate the biochemical changes in tumor cells after Sul-L1-RGD treatment, a pH-sensitive fluorescent probe (BCECF-AM) was used to detect intracellular pH variations. BCECF-AM (the acetoxymethyl ester derivative of BCECF) is widely used for cytoplasmic pH measurement due to its minimal interference from organelles such as lysosomes. Upon entering the cells through passive diffusion, the probe is cleaved by intracellular esterases to form BCECF, which remains inside and emits green fluorescence proportional to the intracellular pH under the same excitation light. Notably, the green fluorescence intensity positively correlates with intracellular pH in tumor cells across a pH range of 6.2 to 9.5. As shown by flow cytometry analysis (Fig. 6*D*), green fluorescence intensity significantly decreased in tumor cells after Sul-L1-RGD treatment, confirming that Sul-L1-RGD can effectively degrade CAIX, resulting in reduced transport of HCO_3_^-^ back into tumor cells and lower intracellular pH of tumor cells.

## Discussion

To degrade membrane-associated protein CAIX, we designed and synthesized some bifunctional compounds according to the IFLD strategy developed in our previous study. Composed of a CAIX recruiting ligand and an integrin recruiting ligand, the bifunctional compounds led to the endocytosis and degradation of the target protein in lysosome by binding to integrin and CAIX on the cell membrane. Among the compounds studied, Sul-L1-RGD proved to be the most effective compound which led to about 50% reduction of CAIX at the cellular level. We speculate that the relatively low degradation ratio is related to the distribution of CAIX in cells, since CAIX is reported to locate both on the cell membrane and in the nucleus. Furthermore, the antiproliferation of Sul-L1-RGD on two tumor cell lines, HT-29 and MDA-MB-231, was demonstrated under hypoxia conditions Therefore, as an efficient CAIX degrader, Sul-L1-RGD has great potential to be applied in cancer therapy with the ability to improve the hypoxic and acidic microenvironment of solid tumors.

PROTACs utilize the UPS to selectively recognize and degrade target proteins, making them an attractive technology for drug development (1, 2, 3). While autophagy-targeting chimeras, autophagosome-tethering compounds, and CMA-targeting chimeras degrade proteins *via* autophagy by transporting them to lysosomes, these methods are ineffective for targeting membrane and extracellular proteins. To overcome this, approaches such as lysosome-targeting chimeras (9, 10, 11), bispecific aptamer chimeras (12), AbTACs, (13) and GlueTACs (14) have been developed, though challenges remain regarding cell permeability and immunogenicity. In this study, we extended the IFLD strategy by linking the high-affinity CAIX ligand Sul-acid to the arginine-glycine-aspartate (Arg-Gly-Asp, RGD) sequence *via* a custom-designed linker, forming Sul-L1-RGD. We demonstrated its efficacy in degrading CAIX, further validating the IFLD strategy. Off-target effects at high doses are a common challenge for targeted degraders, but our findings show that Sul-L1-RGD achieves 50% degradation of the target protein at low concentrations (<25 nM), consistent with our previous work on small-molecule PD-L1 degraders using the IFLD approach. Our data also indicate that degradation is primarily mediated by αvβ3 integrin, with αvβ5 contributing, while αvβ1 is minimally affected, underscoring the strategy's specificity and safety in drug development.

Hypoxia and acidic microenvironments are characteristic of solid tumors. CAIX catalyzes the hydration of carbon dioxide, regulating pH in hypoxic tumors, and plays a critical role in tumor cell proliferation, invasion, and metastasis (25, 26). These conditions also reduce the stability of many chemotherapeutic agents and impair immune cell activity, diminishing anticancer efficacy (27, 28). Combining CAIX inhibitors with chemotherapy has shown improved tumor treatment outcomes (33). Our study demonstrates that Sul-L1-RGD significantly inhibits tumor cell growth, and its combination with immune checkpoint inhibitors also proved to enhance cancer therapy (34). Additionally, given the ease of synthesis, small size, nonimmunogenicity, and controllable pharmacological properties of IFLD-based degraders, modifying Sul-L1-RGD to incorporate immune checkpoint ligands (like using BMS-8 as a high-affinity PD-L1 ligand) could enable dual-target degradation, enhancing immune cell infiltration and cytotoxicity in tumors and presenting a promising therapeutic strategy.

## Experimental procedures

### Materials

Sodium ascorbate, copper sulfate pentahydrate, HATU, DCC, TFA, N,N-diisopropylethylamine (DIEA), and CH_3_CN were purchased from Energy Chemical. Anhydrous N,N-dimethylformamide (DMF), anhydrous dichloromethane, and N-hydroxysuccinimide were purchased from J&K Scientific. Cyclic RGD-alkyne was purchased from Synpeptide Co, Ltd 3-Azido-1-propanamine was purchased from BIOCONE. 9-((tert-Butoxycarbonyl) amino) nonanoic acid, 12-((tert-Butoxycarbonyl) amino) dodecanoic acid, 4-Oxo-4-((5-sulfamoyl-1,3,4-thiadiazol-2-yl) amino) butanoic acid, and Boc-6-aminocaproic acid were purchased from Bidepharm.

### Characterization methods

Analytical RP-HPLC was performed on an Agilent 1260 infinity system equipped with a DAD-UV detector using an Agilent Poroshell 120, EC-C18 column (4.6 mm × 100 mm, 2.7 μm). The RP-HPLC gradient was started at 10% of B (CH_3_CN) and then increased to 100% of B over 20 min (A: 0.1% TFA in water) with a flow rate of 0.5 ml/min. The purity of the compounds used for biological study (>95%) was determined by HPLC. Semipreparative RP-HPLC was performed on the ULTIMAT 3000 instrument (DIONEX). UV absorbance was measured using a photodiode array detector at 220 and 254 nm. The RP-HPLC gradient was started at 1% of B (CH_3_CN) and then increased to 100% of B over 20 min (A: 0.1% TFA in water). High-resolution mass spectra were measured with an ABI Q-star Elite.

### Synthesis of Sul-L1-Azide

Boc-6-aminocaproic acid (20 mg, 86.5 μmol, 1.0 eq) was added to a mixture of DCC (17.8 mg, 86.4 μmol, 1.0 eq) and N-hydroxysuccinimide (10.0 mg, 86.9 μmol, 1.0 eq) in anhydrous DMF (1.5 ml). After the solution was stirred at room temperature for 2 h, 3-azido-1-propanamine (8.7 mg, 86.9 umol, 1.0 eq) and DIEA (143.0 uL, 865.3 umol, 10.0 eq) were added to the above solution. After that, the reaction was stirred for an additional 2 h at room temperature. HPLC was used to track the reaction progress. The mixture was directly purified by RP-HPLC to afford Boc-L1-Azide as a white powder after freeze-drying. The white powder was dissolved in cooled 20% TFA/dichloromethane, after which the reaction was allowed to warm to room temperature and stirred for 1 h. The solvent was evaporated under reduced pressure and the crude deprotected amine was used without further purification. The deprotected amine, Sul-acid(24.2 mg, 86.3 umol, 1.0 eq), and DIEA (143.0 uL, 865.3 umol, 10.0 eq) were dissolved in anhydrous DMF (1 ml), followed by the addition of HATU (39.5 mg, 103.9 umol, 1.2 eq). The reaction was stirred at room temperature for 2 h and monitored by HPLC (Sul-L1-Azide, *t*_R_=8.95 min). The mixture was directly purified by RP-HPLC to afford Sul-L1-Azide as a white powder after freeze-drying (22.5 mg, 47.3 umol, 54%). HRMS (ESI^+^) m/z: calcd for C_15_H_26_N_9_O_5_S_2_ [M + H]^+^ 476.1420, found 476.1493.

### Synthesis of Sul-L1-RGD

Alkyne-cRGD (9.0 mg, 12.6 μmol, 1.0 eq), Sul-L1-Azide (6.0 mg, 12.6 μmol, 1.0 eq), CuSO_4_⋅5H_2_O (1.89 mg, 7.6 μmol, 0.6 eq), and NaVc (10.0 mg, 50.5 μmol, 4.0 eq) were dissolved in a mixture of DMF/H_2_O (1.5 ml, 2:1). The solution was stirred at room temperature for 2 h and monitored by HPLC (Sul-L1-RGD, *t*_R_=7.84 min). The mixture was directly purified by RP-HPLC to afford Sul-L1-RGD as a white powder after freeze-drying (12 mg, 10.1 μmol, 80%). ^1^H NMR (400 MHz, DMSO) δ 13.04 (s, 1H), 8.58 (d, J = 8.4 Hz, 1H), 8.35 (s, 1H), 8.33 (s, 2H), 8.26 (d, J = 7.8 Hz, 2H), 7.97 – 7.91 (m, 4H), 7.67 (s, 1H), 6.93 (d, J = 8.2 Hz, 2H), 6.63 (d, J = 8.2 Hz, 2H), 4.68 (d, J = 6.8 Hz, 3H), 4.38 (d, J = 7.2 Hz, 1H), 4.32 (d, J = 6.9 Hz, 2H), 4.25 (d, J = 5.5 Hz, 2H), 4.14 (dd, J = 15.7, 7.9 Hz, 2H), 3.26 (d, J = 13.2 Hz, 1H), 3.11 (s, 2H), 3.04 – 2.96 (m, 6H), 2.83 – 2.71 (m, 4H), 2.60 (t, J = 7.4 Hz, 3H), 2.47 (t, J = 6.9 Hz, 2H), 2.35 (dd, J = 16.0, 5.9 Hz, 2H), 2.13 (t, J = 7.4 Hz, 2H), 2.05 (d, J = 7.5 Hz, 2H), 1.95 – 1.90 (m, 2H), 1.85 – 1.78 (m, 3H), 1.68 – 1.45 (m, 6H), 1.43 – 1.31 (m, 6H), 1.23 (d, J = 6.7 Hz, 5H). HRMS (ESI^+^) m/z: calcd. for C_48_H_73_ N_18_O_14_S_2_ [M + H]^+^ 1189.4917, found 1189.4990.

### Synthesis of other compounds

Other compounds are synthesized according to the above procedures.

Sul-L2-Azide *t*_R_=16.12, Yield=52%, HRMS(ESI^+^) m/z:calcd. for C_18_H_32_N_9_O_5_S_2_ [M+H]^+^ 518.1890, found 518.1962.

Sul-L2-RGD *t*_R_=9.08, Yield=86%, HRMS(ESI^+^) m/z:calcd. for C_51_H_79_N_18_O_14_S_2_ [M+H]^+^ 1231.5386, found 1231.5459.

Sul-L3-Azide *t*_R_=18.99, Yield=51%, HRMS(ESI^+^) m/z:calcd. for C_21_H_38_N_9_O_5_S_2_ [M+H]+ 560.2359, found 558.2286.

Sul-L3-RGD *t*_R_=10.82, Yield=84%, HRMS(ESI^+^) m/z:calcd. for C_54_H_85_N_18_O_14_S_2_ [M+H]^+^ 1273.5856, found 1273.5929.

### Cell culture

MDA-MB-231 (CL-0150), A549 (CL-0016), and HT-29 (CL-0118) cells lines were purchased from Procell Life Science and Technology. All cell lines were cultured in Dulbecco's modified Eagle's medium (DMEM) (Biological Industries) with 10% fetal bovine serum (Invitrogen) and 1% Pen-Strep solution (Biological Industries). And all of them were cultured in an incubator at 37 °C under 5% CO_2_.

### Western blotting analysis of the CAIX levels and integrin **β**3, **β**5, **β**1 levels

MDA-MB-231 cells or HT-29 cells were cultured in twelve-well cell plates (JET BIOFIL) to a density of 70 to 80%. To screen out the optimal CAIX degraders in Sul-L1-RGD, Sul-L2-RGD, and Sul-L3-RGD with different linker lengths, we diluted them to 5 nM, 25 nM, 50 nM, 100 nM with DMEM and co-incubated them with the cells for 8 h. In addition, to determine the optimal concentration of Sul-L1-RGD and Sul-acid, it was diluted to 5 nM, 25 nM, 50 nM, 100 nM with DMEM and co-incubated with the cells for 8 h. To explore the minimum concentration threshold of Sul-L1-RGD–induced CAIX degradation, it was diluted to 0.04 nM, 0.2 nM, 1 nM, 5 nM, 25 nM with DMEM and co-incubated with the cells for 8 h. For different time gradients, Sul-L1-RGD was diluted to 5 nM and added to the cells at different times. To verify the degradation of CAIX was mediated by *α*v*β*3 integrin, high concentration (5 μM) of cRGD was added into the well plate and the cells were cultured at 4 °C for 1 h, then 25 nM Sul-L1-RGD was added to the well and the cells were cultured at 37 °C for 8 h. When verifying the degradation pathway, bafilomycin A1 (100 nM) was co-incubated with the cells 2 h before Sul -L1-RGD was added, and the cells were cultured at 37 °C for additional 8 h. In another well, MG132 (5 μM) was added along with Sul -L1-RGD and the cells were cultured at 37 °C for 8 h.

To study the degradation trend of integrin at different concentrations and times, Sul -L1-RGD was diluted to 5 nM, 25 nM, 50 nM, 100 nM with DMEM and incubated with cells for 8h, or Sul -L1-RGD was diluted to 5 nM and co-incubated with cells for different times. Then the cells were washed with PBS and 50 to 100 μl of SDS lysis buffer (Beyotime) containing the protease inhibitor cocktail (1 mM) was added. After centrifugation at 14,000 rpm for 3 min, the protein samples were boiled for 20 min and then boiled with SDS-PAGE sample loading buffer (5×) for 10 min. The protein samples were separated by 10% SDS-PAGE for electrophoresis and transferred to 0.45 μm polyvinylidene fluoride membrane (Millipore). Then, the membranes were blocked with 5% nonfat powdered milk in PBST buffer (PBS + 0.1% Tween-20) for 2 h at room temperature with slightly shaking. The membranes were respectively incubated overnight with primary antibodies (CAIX antibody, Cell Signaling Technology, rabbit, 1:1000; GAPDH antibody, Proteintech, mouse, 1:10,000) at 4 °C with slightly shaking. Then, the membranes were washed three times (each for 5 min) with PBST buffer. The membranes were incubated with horseradish peroxidase–conjugated anti-rabbit IgG antibodies (1:10,000 dilution) and anti-mouse IgG antibodies (1:5000 dilution) for 1 h at room temperature. At last, the membranes were washed three times (each for 5 min) with PBST buffer, and the Western blot bands were detected by using an electro chemiluminescence Western blotting substrate (Yeasen Biotechnology, CAT: 36208ES60).

### Cell survival experiment (CCK8)

MDA-MB-231 cells and HT-29 cells were inoculated in 96-well plates with approximately 50% to 60% cell density. Sul-L1-RGD and CoCl_2_ solutions (simulated anoxic environment) were diluted to 5 nM and 100 μM with DMEM, respectively. The prepared DMEM medium containing drugs was added into the cell plates at a fixed time point every day and incubated in a 37 °C incubator for three consecutive days. Then the medicated medium was sucked out of the 96-well plate, and the preconfigured CCK8 solution (10 μl CCK8 solution per 100 μl DMEM) was added, 100 μl per well. After incubation in the 37 °C cell incubator for 10 to 30 min, OD value was determined with microplate reader at the wavelength 450 nm. The determination could be finished until OD value of CTL group was between 1 and 1.2; otherwise, the incubation was continued in the 37 °C cell incubator.

### Intracellular pH measurement

The intracellular pH (pH_i_) of cells was measured using flow cytometry with the pH-sensitive fluorescent probe BCECF-AM (Beyotime Biotechnology, S1006). A549 cells were cultured in twelve-well plates (JET BIOFIL) to 70 to 80% confluence. Hundred micromolars of CoCl_2_ was added post cell adhesion and maintained until BCECF-AM treatment. After incubation with 25 nM and 50 nM Sul-L1-RGD for 8 h, the cell suspension was washed with PBS and labeled with 5 μM BCECF-AM for 1 h. The labeled cells were analyzed at an excitation wavelength of 488 nm, and the average fluorescence intensity of A549 cells in the control and Sul-L1-RGD-treated groups was measured.

### Statistics and reproducibility

All experiments were repeated independently with similar results at least three times. The intensity of Western blot bands were quantified using ImageJ (National Institutes of Health). Data analysis was obtained from GraphPad Prism 9 software (GraphPad Software) through the one-way ANOVA with Sidak's multiple comparisons test.

## Data availability

The data that support the findings of this study are available from the authors upon reasonable request.

## Supporting information

Additional HPLC analysis, ^1^H NMR and HRMS spectra (PDF). This article contains supporting information.

## Conflict of interest

The authors declare that they have no conflicts of interest with the contents of this article.

## References

[1] Li X., Song Y. (2020). Proteolysis-targeting chimera (PROTAC) for targeted protein degradation and cancer therapy. J. Hematol. Oncol..

[2] Zeng S., Huang W., Zheng X., Liyan C., Zhang Z., Wang J. (2021). Proteolysis targeting chimera (PROTAC) in drug discovery paradigm: recent progress and future challenges. Eur. J. Med. Chem..

[3] Zhou X., Dong R., Zhang J.Y., Zheng X., Sun L.P. (2020). PROTAC: a promising technology for cancer treatment. Eur. J. Med. Chem..

[4] Hanzl A., Winter G.E. (2020). Targeted protein degradation: current and future challenges. Curr. Opin. Chem. Biol..

[5] Paiva S.L., Crews C.M. (2019). Targeted protein degradation: elements of PROTAC design. Curr. Opin. Chem. Biol..

[6] Dale B., Cheng M., Park K.S., Kaniskan H.U., Xiong Y., Jin J. (2021). Advancing targeted protein degradation for cancer therapy. Nat. Rev. Cancer.

[7] Li Z., Zhu C., Ding Y., Fei Y., Lu B. (2020). ATTEC: a potential new approach to target proteinopathies. Autophagy.

[8] Takahashi D., Moriyama J., Nakamura T., Miki E., Takahashi E., Sato A. (2019). AUTACs: cargo-specific degraders using selective autophagy. Mol. Cell.

[9] Caianiello D.F., Zhang M., Ray J.D., Howell R.A., Swartzel J.C., Branham E.M.J. (2021). Bifunctional small molecules that mediate the degradation of extracellular proteins. Nat. Chem. Biol..

[10] Banik S.M., Pedram K., Wisnovsky S., Ahn G., Riley N.M., Bertozzi C.R. (2020). Lysosome-targeting chimaeras for degradation of extracellular proteins. Nature.

[11] Ahn G., Banik S.M., Miller C.L., Riley N.M., Cochran J.R., Bertozzi C.R. (2021). LYTACs that engage the asialoglycoprotein receptor for targeted protein degradation. Nat. Chem. Biol..

[12] Miao Y., Gao Q., Mao M., Zhang C., Yang L., Yang Y. (2021). Bispecific aptamer chimeras enable targeted protein degradation on cell membranes. Angew. Chem. Int. Ed. Engl..

[13] Cotton A.D., Nguyen D.P., Gramespacher J.A., Seiple I.B., Wells J.A. (2021). Development of antibody-based PROTACs for the degradation of the cell-surface immune checkpoint protein PD-L1. J. Am. Chem. Soc..

[14] Zhang H., Han Y., Yang Y., Lin F., Li K., Kong L. (2021). Covalently engineered nanobody chimeras for targeted membrane protein degradation. J. Am. Chem. Soc..

[15] Pance K., Gramespacher J.A., Byrnes J.R., Salangsang F., Serrano J.-A.C., Cotton A.D. (2023). Modular cytokine receptor-targeting chimeras for targeted degradation of cell surface and extracellular proteins. Nat. Biotechnol..

[16] Wu Y., Lin B., Lu Y., Li L., Deng K., Zhang S. (2023). Aptamer-LYTACs for targeted degradation of extracellular and membrane proteins. Angew. Chem. Int. Ed..

[17] Zheng J., He W., Li J., Feng X., Li Y., Cheng B. (2022). Bifunctional compounds as molecular degraders for integrin-facilitated targeted protein degradation. J. Am. Chem. Soc..

[18] Slack R.J., Macdonald S.J.F., Roper J.A., Jenkins R.G., Hatley R.J.D. (2022). Emerging therapeutic opportunities for integrin inhibitors. Nat. Rev. Drug Discov..

[19] Campbell I.D., Humphries M.J. (2011). Integrin structure, activation, and interactions. Cold Spring Harb. Perspect. Biol..

[20] Nieberler M., Reuning U., Reichart F., Notni J., Wester H.J., Schwaiger M. (2017). Exploring the role of RGD-recognizing integrins in cancer. Cancers (Basel).

[21] Yu Y.P., Wang Q., Liu Y.C., Xie Y. (2014). Molecular basis for the targeted binding of RGD-containing peptide to integrin alphaVbeta3. Biomaterials.

[22] Danhier F., Le Breton A., Preat V. (2012). RGD-based strategies to target alpha(v) beta(3) integrin in cancer therapy and diagnosis. Mol. Pharm..

[23] Supuran C.T., Winum J.-Y. (2015). Carbonic anhydrase IX inhibitors in cancer therapy: an update. Future Med. Chem..

[24] Supuran C.T. (2008). Carbonic anhydrases: novel therapeutic applications for inhibitors and activators. Nat. Rev. Drug Discov..

[25] Becker H.M. (2020). Carbonic anhydrase IX and acid transport in cancer. Br. J. Cancer.

[26] Parks S.K., Chiche J., Pouyssegur J. (2013). Disrupting proton dynamics and energy metabolism for cancer therapy. Nat. Rev. Cancer.

[27] Thiry A., Dogne J.M., Masereel B., Supuran C.T. (2006). Targeting tumor-associated carbonic anhydrase IX in cancer therapy. Trends Pharmacol. Sci..

[28] Winum J.Y., Rami M., Scozzafava A., Montero J.L., Supuran C. (2008). Carbonic anhydrase IX: a new druggable target for the design of antitumor agents. Med. Res. Rev..

[29] Aldera A.P., Govender D. (2021). Carbonic anhydrase IX: a regulator of pH and participant in carcinogenesis. J. Clin. Pathol..

[30] Chu C.Y., Jin Y.T., Zhang W., Yu J., Yang H.P., Wang H.Y. (2016). CA IX is upregulated in CoCl2-induced hypoxia and associated with cell invasive potential and a poor prognosis of breast cancer. Int. J. Oncol..

[31] Chen J.-J., Chou C.-W., Chang Y.-F., Chen C.-C. (2008). Proteasome inhibitors enhance TRAIL-induced apoptosis through the intronic regulation of DR5: involvement of NF-κB and reactive oxygen species-mediated p53 activation. J. Immunol..

[32] Queen A., Bhutto H.N., Yousuf M., Syed M.A., Hassan M.I. (2022). Carbonic anhydrase IX: a tumor acidification switch in heterogeneity and chemokine regulation. Semin. Cancer Biol..

[33] Andreucci E., Ruzzolini J., Peppicelli S., Bianchini F., Laurenzana A., Carta F. (2019). The carbonic anhydrase IX inhibitor SLC-0111 sensitises cancer cells to conventional chemotherapy. J. Enzyme Inhib. Med. Chem..

[34] Kleinendorst S.C., Oosterwijk E., Molkenboer-Kuenen J., Frielink C., Franssen G.M., Boreel D.F. (2024). Towards effective CAIX-targeted radionuclide and checkpoint inhibition combination therapy for advanced clear cell renal cell carcinoma. Theranostics.

