# Supplementary material for: Bifunctional compounds for targeted degradation of carbonic anhydrase IX through integrin-facilitated lysosome degradation
Source: J Biol Chem. 2025 Apr 7;301(5):108482. doi: 10.1016/j.jbc.2025.108482 (PMC12127573; doi:10.1016/j.jbc.2025.108482)
Supplement: Supporting Information [file mmc1.docx]

**Supporting Information**

Bifunctional compounds for targeted degradation of carbonic anhydrase IX through integrin-facilitated lysosome degradation

Wanyi He ^a, 1^, Congli Chen ^a, b, 1^, Runjie Cai ^a, 1^, Jiwei Zheng ^a^, Mengyu Yao ^a^, Joong Sup Shim ^c^, Hang Fai Kwok ^c^, Xiaojun Yao ^d^, Lijing Fang ^a, e,^ *, Liang Chen ^a, e,^ *

*^a^ Institute of Biomedicine and Biotechnology, Shenzhen Institute of Advanced Technology, Chinese Academy of Sciences, Shenzhen 518055 Guangdong, China*

*^b^ School of Pharmacy, Changzhou University, Changzhou, Jiangsu Province 213164, China*

*^c^ Cancer Centre, Faculty of Health Sciences, University of Macau, Avenida da Universidade, Taipa, Macau SAR*

*^d^ Centre for Artificial Intelligence Driven Drug Discovery, Faculty of Applied Sciences, Macao Polytechnic University, Macao 999078*

*^e^ Institute of Biomedicine and Biotechnology, Shenzhen Institute of Advanced Technology, Chinese Academy of Sciences, Shenzhen 518055 Guangdong, China*

[*lj.fang@siat.ac.cn*](mailto:lj.fang@siat.ac.cn) *and liang.chen@siat.ac.cn*

**Table of Contents**

^1^H NMR Spectra……………………………………………………………………………………1

HRMS Spectra…………………………………………………………………………………...…2

HPLC Analysis…………………………………………………………………………………..…5

**^1^H NMR Spectra**

**
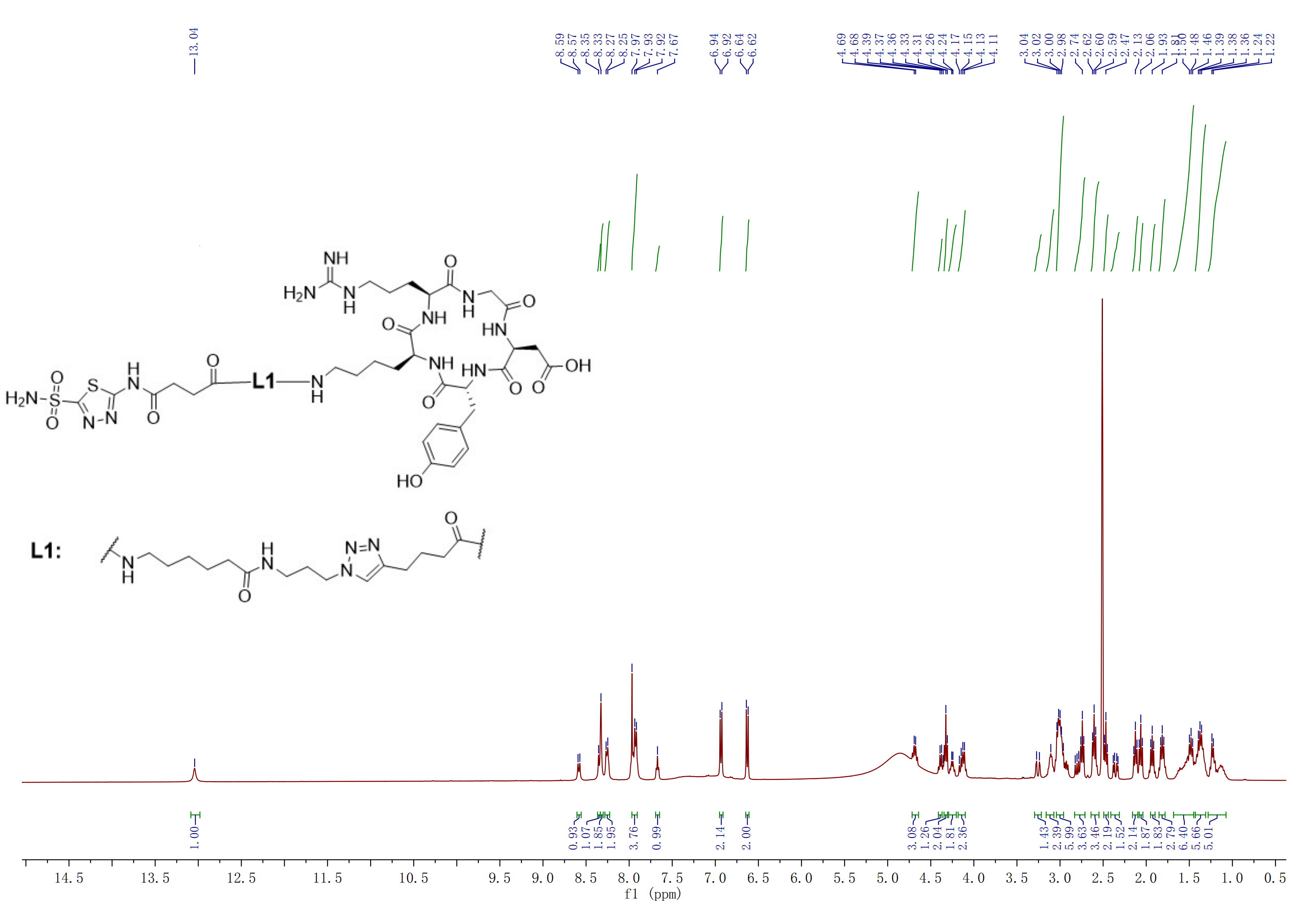
**

Figure S1. ^1^H NRM spectrum of Sul-L1-RGD (DMSO-d_6_)

**HRMS Spectra**


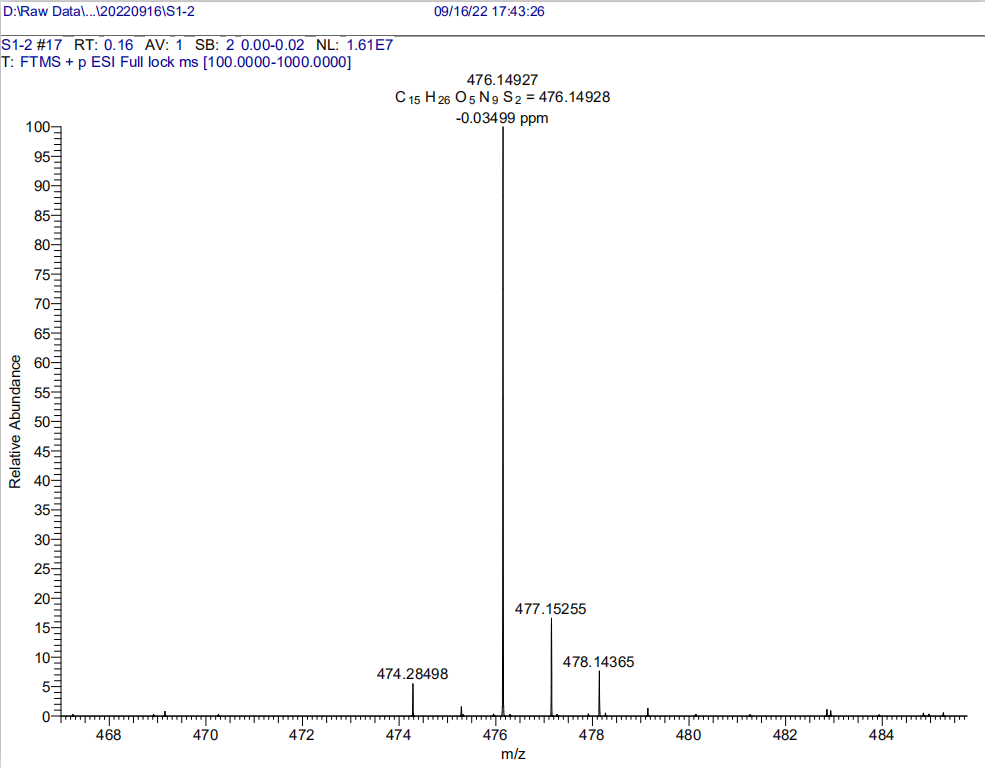


Figure S2. HRMS spectrum of Sul-L1-Azide


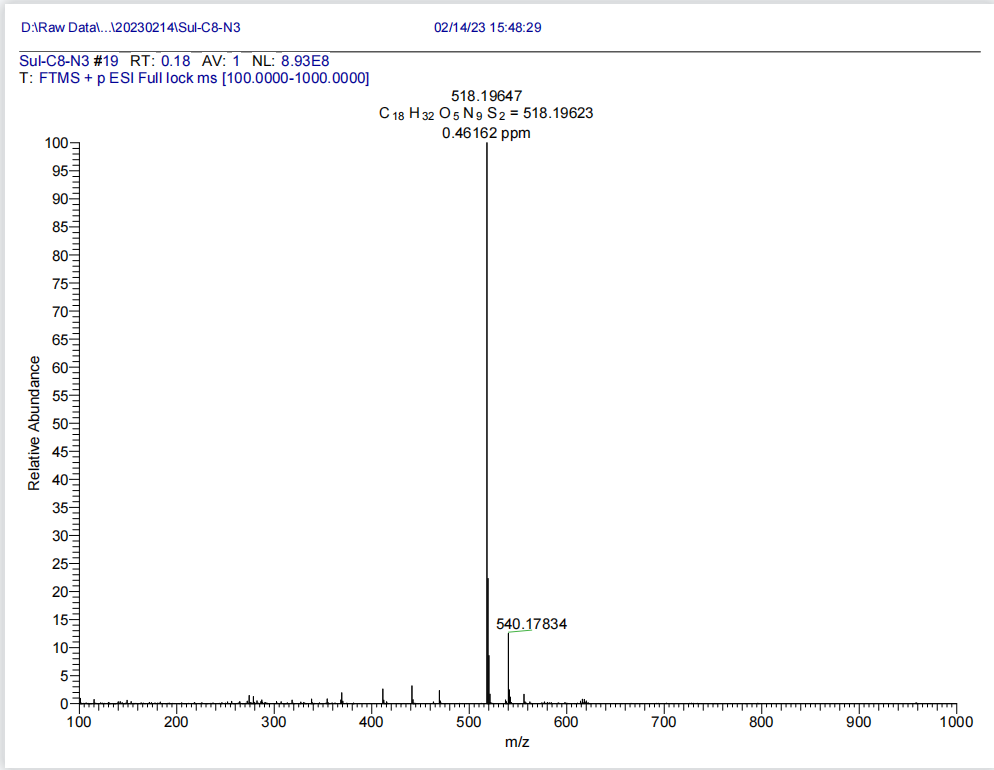


Figure S3. HRMS spectrum of Sul-L2-Azide


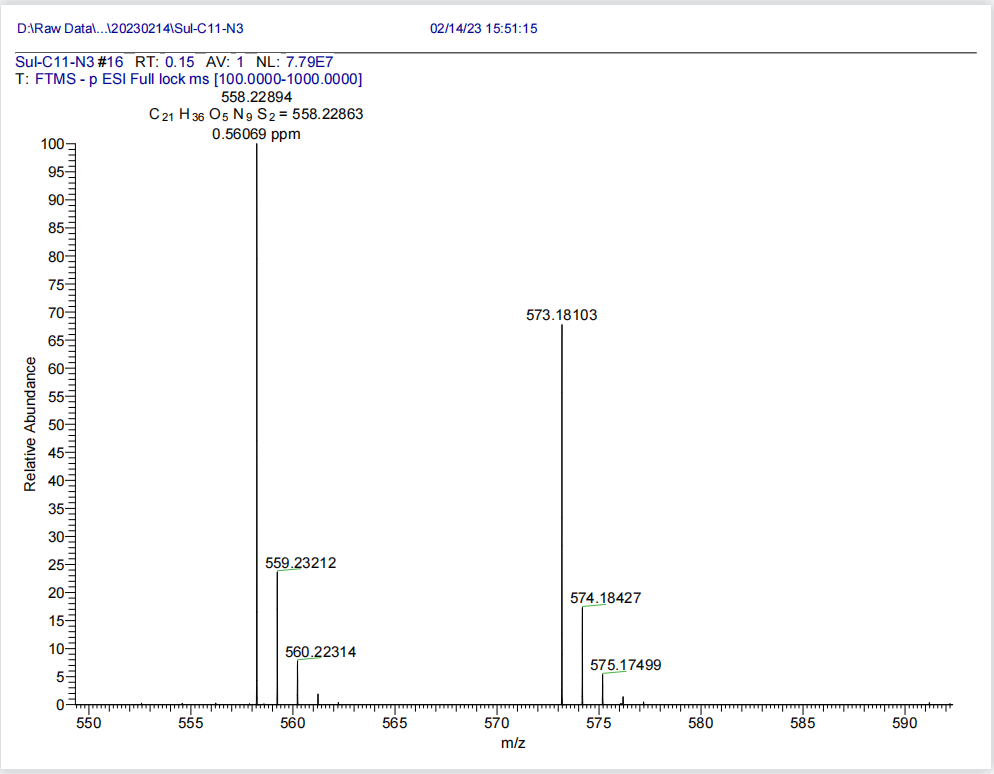


Figure S4. HRMS spectrum of Sul-L3-Azide


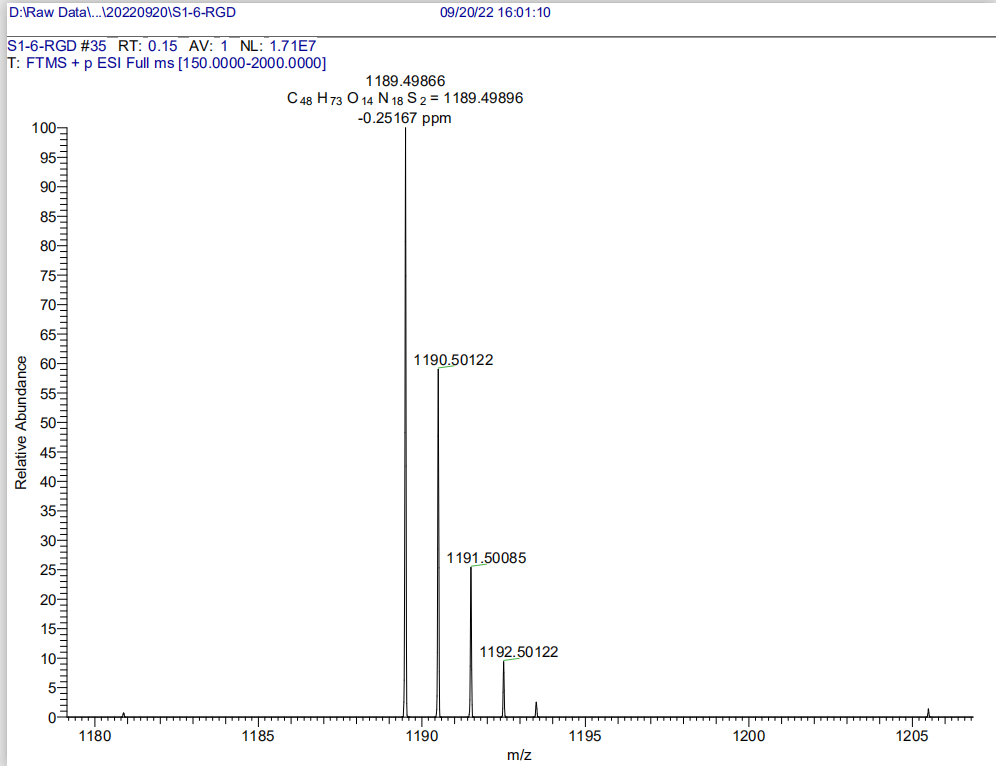


Figure S5. HRMS spectrum of Sul-L1-RGD


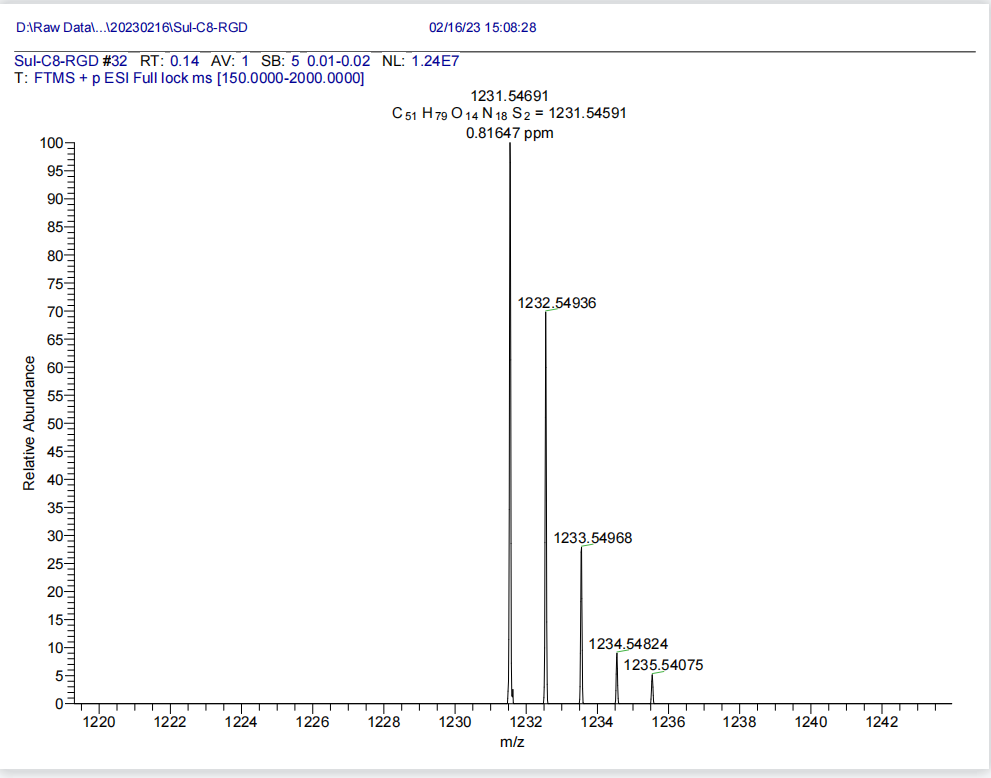


Figure S6. HRMS spectrum of Sul-L2-RGD


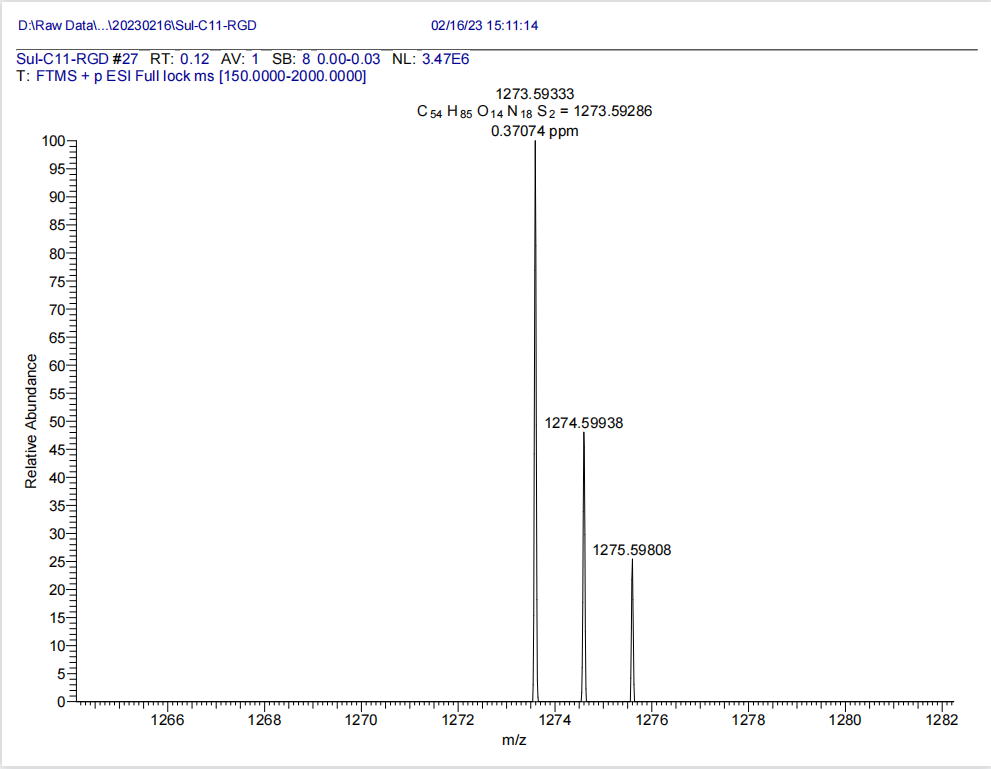


Figure S7. HRMS spectrum of Sul-L3-RGD

**HPLC Analysis**


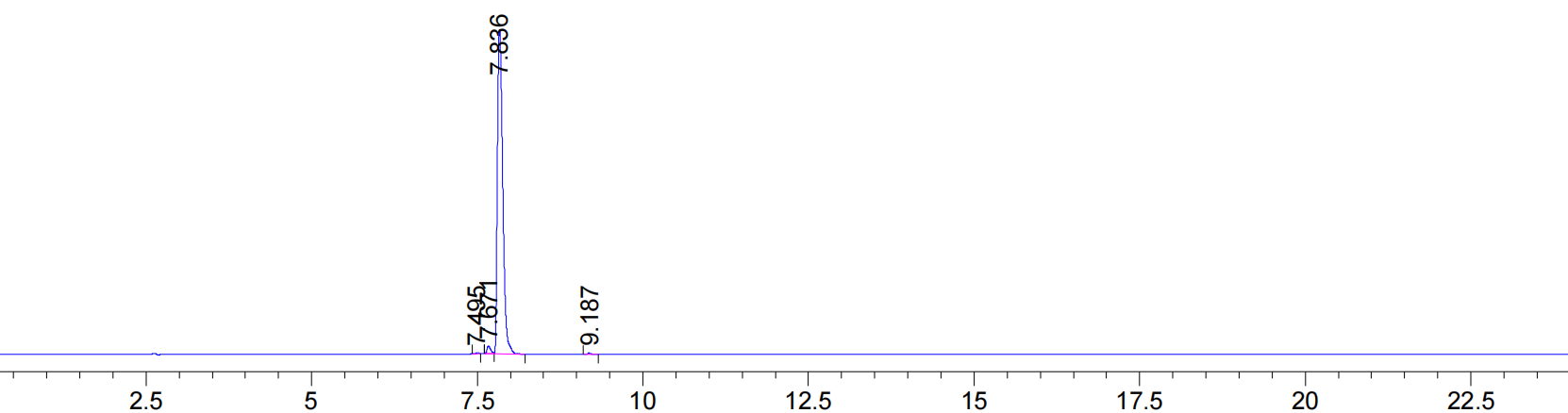


| Peak # | Retention Time (min) | Height (mAU) | Area (mAU*s) | Area% |
| --- | --- | --- | --- | --- |
| 1 | 7.495 | 4.65398 | 21.00118 | 0.2340 |
| 2  3  4 | 7.671  7.836  9.187 | 37.90839  1558.00415  6.79188 | 147.75377  8778.78125  28.21955 | 1.6461  97.8055  0.3144 |
| Totals | | 1607.35839 | 8975.75574 |  |

Figure S8. HPLC analysis of Sul-L1-RGD (254 nm Result)


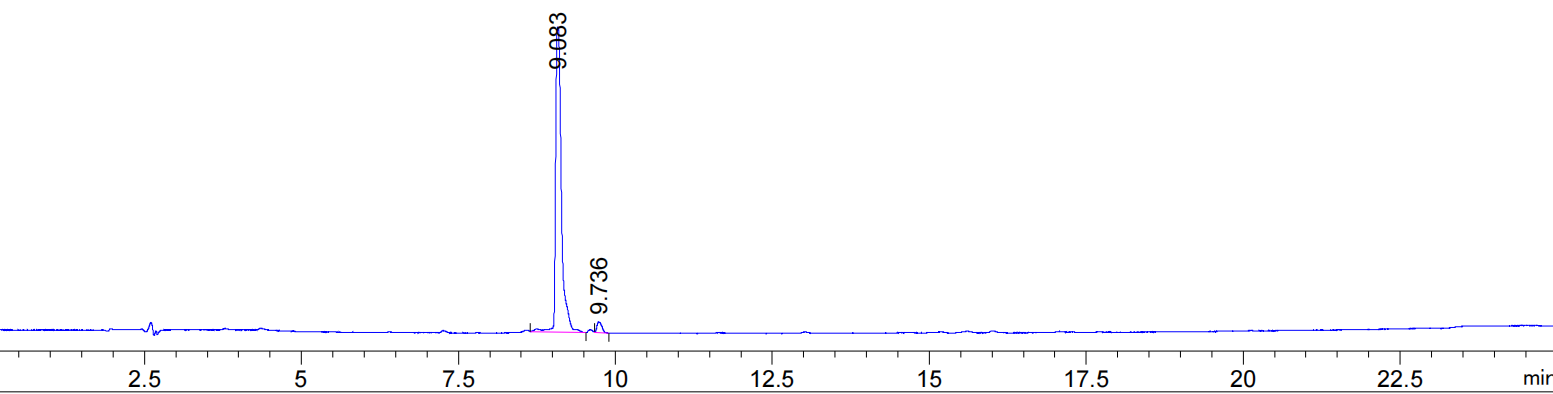


| Peak # | Retention Time (min) | Height (mAU) | Area (mAU*s) | Area% |
| --- | --- | --- | --- | --- |
| 1 | 9.083 | 134.00449 | 860.00537 | 97.0056 |
| 2 | 9.7336 | 4.85665 | 26.54678 | 2.9944 |
| Totals | | 138.86114 | 886.55215 |  |

Figure S9. HPLC analysis of Sul-L2-RGD (254 nm Result)


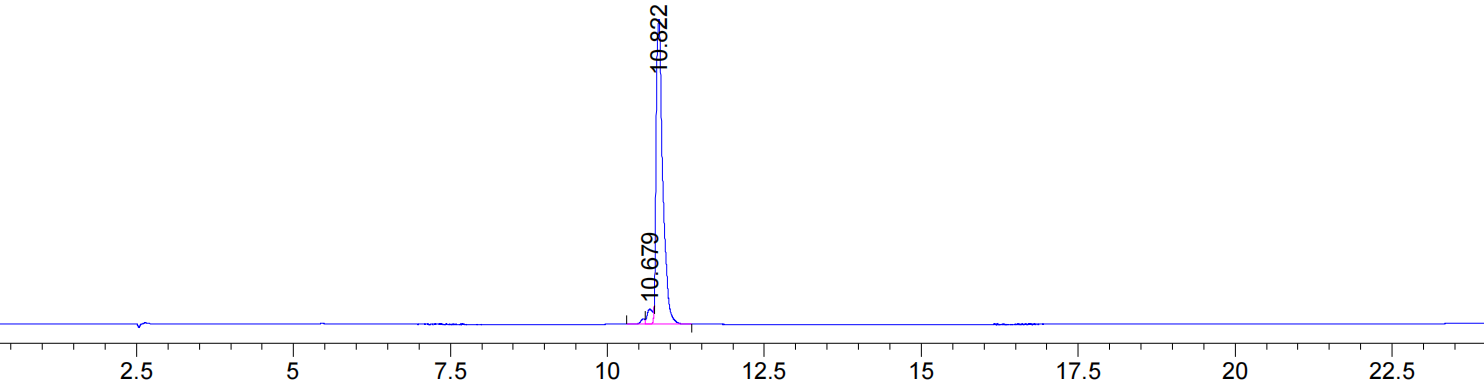


| Peak # | Retention Time (min) | Height (mAU) | Area (mAU*s) | Area% |
| --- | --- | --- | --- | --- |
| 1 | 10.679 | 44.11445 | 261.74500 | 3.7751 |
| 2 | 10.822 | 895.52148 | 6671.80518 | 96.2249 |
| Totals | | 939.63594 | 6933.55017 |  |

Figure S10.HPLC analysis of Sul-L3-RGD (254 nm Result)
